# BRCA2 germline mutation carrier with five malignancies: a case report

**DOI:** 10.1186/s13053-024-00302-7

**Published:** 2024-12-19

**Authors:** Elena Su, Yann Christinat, Thomas McKee, Silvia Azzarello-Burri, Wolfram Jochum, Stefanie Fischer, Christian Rothermundt

**Affiliations:** 1 Department of Gynecology, Cantonal Hospital St. Gallen, Rorschacher Strasse 95, St. Gallen, 9007 Switzerland; 2 Department of Clinical Pathology, Geneva University Hospitals, Rue Gabrielle-Perret- Gentil 4, Geneva, 1206 Switzerland; 3 Aurigen SA, Avenue de Sévelin 18, Lausanne, 1004 Switzerland; 4 Institute of Pathology, Cantonal Hospital St. Gallen, Rorschacher Strasse 95, St. Gallen, 9007 Switzerland; 5 Department of Medical Oncology and Haematology, Cantonal Hospital St. Gallen, Rorschacher Strasse 95, St. Gallen, 9007 Switzerland; 6 Department of Medical Oncology, Cantonal Hospital Lucerne, Spitalstrasse 16, Lucerne, 6000 Switzerland

**Keywords:** BRCA2 mutation, APC, Genetic testing, Whole-genome sequencing, Breast cancer, Renal cell carcinoma, Liposarcoma, Myeloproliferative neoplasia

## Abstract

**Background:**

BRCA2 germline mutations are known to predispose carriers to various cancer types, including breast, ovarian, pancreatic and prostate cancer. An association with melanoma has also been reported. However, the full tumour spectrum associated with *BRCA2* mutations, particularly in patients with other concurrent pathogenetic mutations, is unexplored.

**Case presentation:**

We present a 70-year-old female patient with a pathogenic *BRCA2* c.5946del variant. Over a period of 15 years, she has developed two independent breast cancers, well-differentiated liposarcoma, clear cell renal cell carcinoma and myeloproliferative neoplasia. This unusual tumour spectrum and the staggered occurrence of these tumours required multiple rounds of genetic testing and led to a delayed diagnosis of the *BRCA2*-associated tumour predisposition. In addition to the *BRCA2* mutation, extended germline testing revealed an *APC* c.3920T > A variant and variants of unknown significance in the *BRIP1* and *ATR* genes. The molecular analysis of the tumours revealed distinct profiles with differences in HRD status and in copy number variations, indicating no common origin.

**Conclusions:**

Our case study revealed that the pathogenic *BRCA2* c.5946del germline variant can be associated with an unusual tumour spectrum, which may lead to a delayed diagnosis of a hereditary tumour predisposition. Thus, upfront genetic testing using large multigene panels or whole-genome sequencing in encouraged, especially in cases with a prominent family history.

## Background

Mutations in the *BRCA1* and *BRCA2* genes have long been known to predispose individuals to certain malignancies, such as female breast and ovarian cancer [1, 2]. Associations of *BRCA* mutations with pancreatic and prostate cancer have also been established [3–5]. A trend toward melanoma has been reported by multiple groups but remains controversial [3, 6]. A recent study by Momozawa et al. suggested that the range of cancer types associated with mutated and possibly pathogenic forms of *BRCA1* and *BRCA2* might be broader, including biliary tract cancer, gastric cancer and oesophageal cancer [7]. A germline mutation in the *APC* gene, on the other hand, has long been associated with familial adenomatous polyposis [8, 9]. It has also been reported that *APC* mutations contribute to chromosomal instability [10, 11].

In this report, we present the case of a female *BRCA2* mutation carrier who was diagnosed with five different malignancies: two independent breast cancers, well-differentiated liposarcoma, clear cell renal cell carcinoma (ccRCC) and therapy-related myeloproliferative neoplasia. While the association between *BRCA2* mutations and breast cancer has been well studied, few or no studies have investigated the associations between BRCA2 mutations and other malignancies present in our case. It has been reported that first- and second-degree relatives of *BRCA* mutation carriers have increased standardized incidence ratios for kidney cancer [12]. Similarly, Momozawa et al. also observed an association of pathogenic variants in *BRCA2* with kidney cancer [7]. In contrast, a meta-analysis revealed no increased risk for kidney cancer in the presence of *BRCA* mutations [13]. To date, we have found no literature on the association of *APC* mutations with kidney cancer or of *BRCA1*, *BRCA2* or *APC* mutations with liposarcoma of any kind.

## Case presentation

Our patient is a 70-year-old female carrier of a *BRCA2* mutation. Over a period of 15 years, she has developed five different malignancies. In 2006, a well-differentiated liposarcoma of the right thigh (atypical lipomatous tumour (ALT)) was first diagnosed and surgically removed. In 2008, a ccRCC of the right side (initially pT1 cN0 cM0 G2) was detected and surgically removed. She then developed a right adrenal metastasis, which was surgically removed in 2010, and a solitary bone metastasis in the second left rib was resected in 2016. In 2010, a poorly differentiated invasive ductal breast carcinoma was found in the right external inferior quadrant (initially pT1c (11 mm) pN0 (sn)(0/4) M0 G3). In 2018, therapy-associated myeloproliferative neoplasia of the primary myelofibrosis type was diagnosed in the prefibrotic phase. Most recently, in 2021, a type of breast carcinoma, no special type (NST), was found in the lower left interior quadrant (initially pT2 (37 mm) pN1 (2/2) M0 R0 G3).

**Fig. 1:**
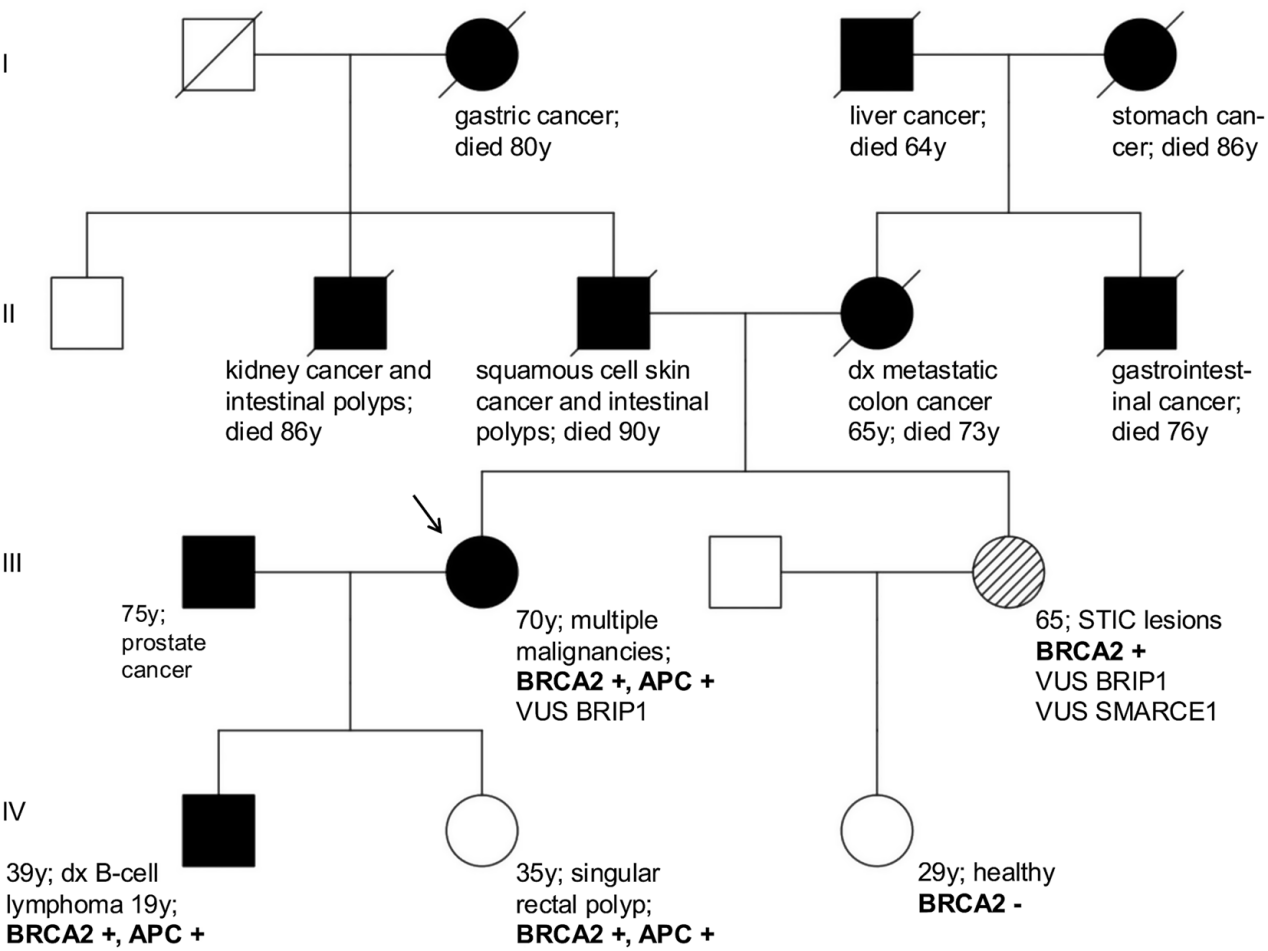
Three generational pedigrees. Square, male; circle, female; strikethrough, deceased; black fill, cancer; white fill, no relevant disease; stripes, STIC lesions. The current age or age at death was known. Germline *BRCA2* and *APC* positive (+) and negative (−) genetic test results are indicated. VUS mutations are indicated

The patient has a prominent family history of different malignant diseases (see Fig. 1). Her family was Northern European and likely of Ashkenazi Jewish descent. Her son was diagnosed with cutaneous marginal zone B-cell lymphoma at the age of 19 years; in her daughter, a singular rectal polyp was found and removed. Her sister underwent prophylactic removal of the adnexa. In the biopsy, serous tubal intraepithelial carcinoma (STIC) lesions, precursor cells of an ovarian carcinoma, and P53 lesions were found in both fallopian tubes.

The patient’s mother was diagnosed with colon cancer at 65 years of age, and she was also found to have bladder cancer and lung cancer (possibly metastatic colon cancer). Her maternal uncle died of a gastrointestinal tumour at the age of 76, and the maternal grandfather died of liver cancer at the age of 64. A niece of this grandfather died of pancreatic cancer at 72 years of age, as did her daughter at the age of 45. The family history was also remarkable on the side of our patient’s father: A squamous cell skin cancer was detected in the father at an older age, and he was also known to have intestinal polyps. An uncle died due to kidney cancer at the age of 86 years and had polyps. A cousin on the father’s side was also diagnosed with intestinal polyposis, and his brother died with prostate cancer at the age of approximately 57 years. The paternal grandmother died at 80 years of age from gastric cancer.

### Genetic testing

Owing to the unusual tumour spectrum and the staggered occurrence of these tumours, multiple rounds of genetic testing were performed. After the ALT diagnosis, a genetic analysis of the *TP53* gene (sequencing and MLPA analysis) revealed normal results, ruling out possible Li‒Fraumeni syndrome. Subsequently, several other genes associated with sarcoma, intestinal cancer, kidney cancer and breast cancer (*APC*,* MUTYH*,* MSH2*,* MLH1*,* MSH6*,* PMS2*,* STK11*,* SMAD4*,* BMPR1A*,* PTEN*,* TP53*,* CDH1*,* PALB2*,* RAD51C*,* RAD51D*,* SDHB*,* SDHC*,* SDHD*,* and VHL*) were analysed, and an *APC* gene (NM_000038.6) variant (c.3920T > A, p.(Ile1307Lys) was detected. This variant is reported in approximately 5–10% of the Ashkenazi Jewish population and confers a 1.5–1.75-fold increased risk for colorectal cancer [14]. Only after the occurrence of the second breast cancer exome sequencing and analysis of a panel of 97 hereditary tumour syndrome-associated genes was performed on DNA extracted from blood, saliva, and hair. This analysis revealed the *BRCA2* (NM_000059.4) heterozygous pathogenic variant c.5946del, p. (Ser1982Argfs*22) and 2 heterozygous variants of unknown significance (VUS) in the *BRIP1* gene (NM_032043.3): c.1444 A > G, p. (Ile482Val) and in the *ATR* gene (NM_001184.4): c.6226 C > G, p. (Leu2076Val). The patient underwent prophylactic adnexectomy with no evidence of malignancies in the fallopian tubes.

Both children of the patient carry the pathogenic *BRCA2* variant c.5946del, p. (Ser1982Argfs*22) and the *APC* variant c.3920T > A, p. (Ile1307Lys). Similarly, the heterozygous pathogenic *BRCA2* variant c.5946del, p. (Ser1982Argfs*22) and the VUS in the *BRIP1* gene heterozygous c.1444 A > G, p. (Ile482Val) were also found in the patient’s sister. The sister also carries another VUS in the *SMARCE1* gene c.1210 A > G, p.Ile404Val. Her daughter tested negative for *BRCA* mutations.

### HRD status and copy number variations

**Fig. 2:**
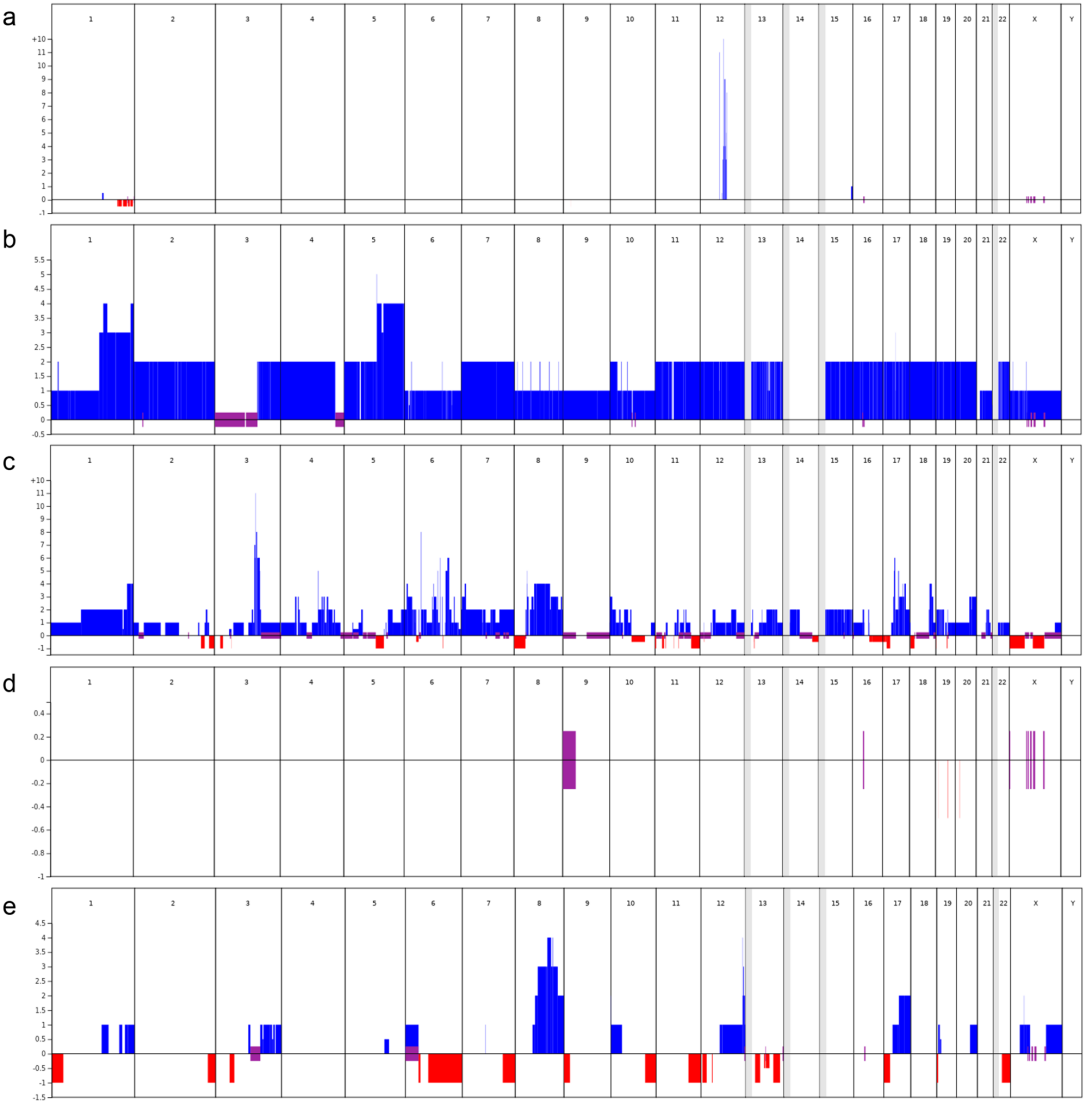
Copy number variations. Blue, amplification or gain; red, loss; purple, loss of heterozygosity (LOH); grey, not covered by Oncoscan. (**a**) Well-differentiated liposarcoma of the right thigh, 2006; (**b**) clear cell renal cell carcinoma, 2008; (**c**) poorly differentiated invasive ductal breast carcinoma, 2010; (**d**) therapy-associated myeloproliferative neoplasia of primary myelofibrosis type, 2018; (**e**) breast carcinoma, no special type (NST), 2021

Samples of all five tumours were tested for homologous recombination deficiency (HRD) and copy number variations via the Geneva HRD test (Thermo Fisher Oncoscan assay) (see Fig. 2; Table 1).

**Table 1:** Results of the Geneva HRD assay

Tumour, year of diagnosis	HRD Status (nLST)	Copy number				Other relevant alterations
		BRCA2	APC	BRIP1	ATR	
Well-differentiated liposarcoma (ALT), 2006	Neg. (1)	No alteration	No alteration	No alteration	No alteration	Focal amplification of 12q14-15 (*MDM2*, *CDK4*) [15]
Clear cell renal cell carcinoma, 2008	Neg. (0)	4 copies	5–6 copies (on a CN breakpoint)	4 copies	4 copies	LOH of 3p arm (*VHL*, *PBRM1*, *BAP1*) [16], 5 copies of *MDM4* Tetraploid tumour
Poorly differentiated invasive ductal breast carcinoma, 2010	Pos. (30.5)	LOH (2 copies)	1 copy	5/6 copies	LOH (3 copies)	Heterozygous loss of *TP53* [17], 4 copies of *MDM4*, triploid tumour
Therapy-associated myeloproliferative neoplasia, 2018	Neg. (0)	No alteration	No alteration	No alteration	No alteration	LOH of 9p arm (*JAK2*) [18]
Breast carcinoma (NST), 2021	Pos. (25)	LOH (1 copy)	No alteration	4 copies	2.5 copies	Heterozygous loss of *TP53*

The ALT displays focal amplification of 12q14-15 (representing *MDM2* and *CDK4*) with an otherwise stable genome and no copy number alterations in *BRCA2*,* BRIP1*,* ATR* or *APC*. In the ccRCC specimen, copy gains in *BRCA2*, *BRIP1*,* ATR* and *APC* can be observed within the context of a tetraploid tumour. Notably, the *APC* gene is on a breakpoint, which might indicate that one allele is lost while the other is amplified. The poorly differentiated invasive ductal breast carcinoma is a triploid tumour with copy gains in *BRIP1* and *ATR* and a high number of alternations. The same tumour also displays a loss of heterozygosity (LOH) in the *APC*. The breast cancer NST displays many alternations, including a loss of *BRCA2* but no loss of *APC*. The therapy-associated myeloproliferative neoplasia of the primary myelofibrosis type does not show any significant alterations.

## Discussion and conclusions

The tumours in this patient fall outside the typical disease spectrum currently associated with *BRCA2* germline mutations. This unusual tumour spectrum may be due to the additional germline variants that were detected in this patient. The detected *APC* c.3920T > A variant and the *BRIP1* and *ATR* VUS may have altered the characteristic tumour spectrum associated with pathogenic *BRCA2* variants. There is a report on ClinVar about the specific *BRIP1* germline mutation found in our patient, and this case is also associated with hereditary breast cancer [19]. For the *ATR* mutation, there is also a report with uncertain significance [20]. Thus, although both the *BRIP1* and *ATR* genes are involved in DNA repair, there is no evidence that these variants have an impact on carcinogenesis in our patient based on copy number variations. It is also not clear whether all five malignancies can be attributed to the detected mutations or whether there might be an additional hereditary cause based on the positive family history of both parents. As the patient’s parents did not undergo genetic counselling, however, it is unknown whether they carried more mutations. Thus, it is not clear from which parental side the *BRCA2* variant and *APC* variants were transmitted. While the two breast cancers fit the spectrum of a *BRCA2 *mutation, the patient did not have gastrointestinal polyps, as might be suggested in the context of her family history of multiple intestinal polyps. It also remains unclear whether yet unexplored environmental factors could have had an impact on the various cancers of the patient and her family.

The molecular analysis of the tumours revealed distinct profiles and did not suggest a common origin. In terms of copy number variation, there are few common breakpoints, and the LOH status of several regions is, timewise, not consistent with a clonal evolution. The first two tumours, ALT and ccRCC, are characterized by typical tumour profiles and are driven by copy number alternations [15, 16]. While the liposarcoma is likely driven by the amplification of *MDM2* and *CDK4*, the ccRCC is likely driven by the loss of 3p and multiple genetic alternations. It seems that both tumours have developed regardless of the mutations found, except that both tumours are driven by copy number changes and that both genes are linked to copy number alterations. The two breast cancers, however, are both HRD positive and have a loss of at least one copy number of *BRCA2*. In the 2010 breast cancer, there was a total loss of *BRCA2*, which might explain why the tumour was HRD positive. In the 2021 breast cancer, there was only one remaining copy which might contain the *BRCA2* mutation. Tumour genotyping was not performed to definitively determine whether the retained alleles are mutant or wild type, as the primary aim of the Oncoscan analysis was to assess a potential common origin rather than to fully characterize the tumours. However, the HRD-positivity observed in both breast tumours strongly supports functional impairment of the homologous recombination pathway. Interestingly, *APC* is lost in the first breast cancer case but is still functional in the second case, which coincides with the fact that only the first breast cancer case is polyploid. The alterations in the 2021 breast cancer samples, in contrast to those in the 2010 sample, appear to be in the form of aneuploidy in addition to HRDness. Most likely, owing to the therapy-associated nature of myeloproliferative neoplasia of primary myelofibrosis type, there are no significant alterations in copy number.

It could be hypothesized that the extra copies of chromosomal arm 1q in some of the cancers might have led to the downregulation of p53 via MDM4 gains, as recently described by Girish et al. [21]. Interestingly, *APC* and *TP53* mutations have also been linked to aneuploidy, which is observed in the kidney cancer and the 2011 breast cancer [10, 11]. There is no loss of *BRCA2* or *APC* in the kidney cancer despite the germline mutations, indicating that these mutations might not drive kidney cancer. The tumour, however, is tetraploid with a loss of heterozygosity on the 3p arm. This is common, especially in clear cell kidney cancer, as many drivers of kidney cancer are located on this arm (*VHL*, *PBRM1* and *BAP1*) [22]. As the molecular analysis of the tumours does not suggest a common origin, it could also be hypothesized that the *BRCA2* mutation – alone or in combination with the *APC* variant – has fragilized the genome. This chromosomal instability, in turn, might have increased the propensity of copy number-associated tumours to develop. This notion would explain why the detected copy number variants do not occur in every or most tumours. Interestingly, not all tumours have an HRD phenotype, which would be expected if they would have evolved on the basis of a *BRCA2* mutation.

While our patient did not have gastrointestinal polyps, the *APC* p.I1307K variant has previously been reported to predispose to other cancers than just intestinal polyps. Lenosh et al. investigated the overall cancer risk among *APC* I1307K polymorphism carriers in an Israeli cohort and showed an increased prevalence of breast and skin cancers in female carriers [23]. Interestingly too, another study described a moderately increased risk of developing renal cancer for either gender of *APC* I1307K carriers with Ashkenazi Jewish ancestry [24]. Previous studies on the coexistence of the *APC* I1307K variant with *BRCA* mutations showed that *APC* I1307K increased the penetrance of *BRCA* mutations for breast cancer [25]. Coinheritance of germline mutations in the *BRCA2 gene* and *APC* variants has once been described before. Dolkar et al. reported a male patient with colonic adenocarcinoma [26]. Sequencing identified a deleterious c.8297delC variant in the *BRCA2* gene and a pathogenic substitution mutation at nucleotide position 1213 in exon 9 of *APC* gene resulting in a premature stop codon (p.R405X). It was not apparent which mutation caused the colon cancer and whether the germline mutations interacted and concurrently predisposed the affected individual to an increased risk of certain cancers.

The coinheritance of germline mutations in the *BRCA2* and the *APC* genes appears to be a very rare event in the current literature. However, with the growing implementation of next-generation sequence-based panel testing for multiple genes involved in tumour predisposition syndromes, the detection of relevant variants will become more frequent. As already noted, the detected *APC* variant is not a highly penetrant mutation with typical polyposis, but a polymorphism that is described in 5–10% of the Ashkenazi Jewish population. *BRCA* founder mutations are also relatively common in this population group [27]. Given this, it can be hypothesized that the coexistence of an *APC* polymorphism and a *BRCA* mutation may not be exceedingly rare. This underscores the importance of informing affected individuals about any additional cancer risks they may face in such cases. Providing optimal monitoring and guidance, as recommended by Valle et al. is crucial for offering these patients the best possible care [28].

Our study is the first report that we are aware of showcasing a female patient carrying both a *BRCA2* and *APC* germline mutation and presenting with multiple malignancies. The unusual tumour spectrum of this patient required multiple rounds of germline testing and led to a delayed diagnosis of the *BRCA2*-associated tumour predisposition. We thus encourage timely and extended genetic testing to detect other potentially relevant mutations. Especially in patients with hereditary cancer, collecting a detailed family history and performing large multigene panels or whole-genome sequencing could be favoured.

## Methods

### Whole-exome sequencing

The coding exons, including approx. 20 bp of the flanking intronic regions, of the following genes were analysed by NGS (WES Library Prep: Gen^®^ Exome Research Panel v2.0 (IDT)); sequenced with NovaSeq 6000 and S1 Reagent Kit (300 cycles) (Illumina Inc.), analysed with NxClinical software; approximately 95% of the tested exons had coverage of 100%: 30 or more reads): *ACD*,* AKT1*,* APC*,* ATM*,* AXIN2*,* BAP1*,* BLM*,* BMPR1A*,* BRCA1*,* BRCA2*,* BRIP1*,* CASR*,* CDC73*,* CDH1*,* CDK4*,* CDKN1B*,* CDKN2A*,* CHEK2*,* CYLD*,* CTRC*,* DDB2*,* DICER1*,* DKC1*,* EPCAM*,* ERCC1*,* ERCC2*,* ERCC3*,* ERCC4*,* ERCC5*,* FANCA*,* FANCB*,* FANCC*,* FANCE*,* FANCF*,* FANCG*,* FANCI*,* FANCL*,* FANCM*,* FH*,* GALNT12*,* GREM1*,* HRAS*,* KIF1B*,* KIT*,* MAX*,* MC1R*,* MEN1*,* MITF*,* MLH1*,* MLH3*,* MSH2*,* MSH3*,* MSH6*,* MUTYH*,* NF1*,* NTHL1*,* PALB2*,* PDGFRA*,* PMS1*,* PMS2*,* POLD1*,* POLE*,* POT1*,* PRKAR1A*,* PRSS1*,* PTCH1*,* PTEN*,* RAD51C*,* RAD51D*,* RB1*,* RECQL4*,* RET*,* RNF43*,* RPS20*,* SDHA*,* SDHAF2*,* SDHB*,* SDHC*,* SDHD*,* SLX4*,* SMAD4*,* SPINK1*,* SPRED1*,* STK11*,* SUFU*,* TERF2IP*,* TERT*,* TMEM127*,* TP53*,* TRIM37*,* TSC1*,* TSC2*,* VHL*,* WRN*,* XPA*,* XPC*,* XRCC2.*

### HRD and copy number analysis

The samples were analysed with the Oncoscan FFPE Assay Kit (cat. 902695; Thermo Fisher Scientific) following the manufacturer’s instructions. The arrays were stained at the GeneChip Fluidics Station (Thermo Fisher Scientific) and scanned via a Gene Chip scanner (Thermo Fisher Scientific, Waltham, Massachusetts, United States). CEL files (Affymetrix DNA microarray image analysis software output) generated from the scanned array image were converted to Oncoscan array data (OSCHP files) and analysed via Chromosome Analysis Suite (ChAS) software (version 4.0; Thermo Fisher) with reference files NetAffxGenomicAnnotations.Homo_sapiens, hg19; NA33.r1. The copy number variation segments were evaluated manually in ChAS. The HRD score (nLST) was computed in R with the OncoscanR package [29].

## Data Availability

All the data generated or analysed during this study are included in this published article. Further inquiries can be directed to the corresponding author.

## Abbreviations

VUS: Variants of unknown significance
ccRCC: Clear cell renal cell carcinoma
ALT: Atypical lipomatous tumour
NST: No special type
STIC: Serous tubal intraepithelial carcinoma
HRD: Homologous recombination deficiency
LOH: Loss of heterozygosity
